# Oligometastases/Oligo-Recurrence of Lung Cancer

**DOI:** 10.1155/2013/438236

**Published:** 2013-02-14

**Authors:** Yuzuru Niibe, Joe Y. Chang, Hiroshi Onishi, Joseph Salama, Takao Hiraki, Hideomi Yamashita

**Affiliations:** ^1^Department of Radiology and Radiation Oncology, Kitasato University School of Medicine, 1-15-1 Kitasato, Minami-ku, Kanagawa, Sagamihara 252-0374, Japan; ^2^Department of Radiation Oncology, Yamanashi University School of Medicine, Yamanashi, Japan; ^3^Department of Radiation Oncology, Duke University, Durham, NC, USA; ^4^Department of Radiation Oncology, Yamanashi University School of Medicine, Yamanashi, Japan; ^5^Department of Radiology, Okayama University Medical School, Okayama, Japan; ^6^Department of Radiology, The University of Tokyo Hospital, Tokyo, Japan

Metastasis or recurrence of cancer has been considered as representing a near terminal life stage. As a result, for a long time, cancer patients with metastasis or recurrence have been classified as one group and treated using only systemic therapy. However, recent advances in cancer therapy have dramatically improved both local and systemic therapies. The concept of oligometastases was proposed by Hellman and Wechselbaum in 1995 [1] and revised by Niibe et al. in 2006 as oligo-recurrence [2]. These notions represent the first classification of metastasis or recurrence to identify subgroups for achieving long-term survival or even cure.

Oligometastases are defined as 1–5 distant metastases that can be treated by local therapy to achieve long-term survival or cure. The most important prognostic factor for oligometastases is the status of the primary lesion [3, 4]. Niibe et al. proposed the concept of oligo-recurrence to overcome this problem. Oligo-recurrence is thus defined as 1–5 distant metachronous metastases that can be treated by local therapy, under conditions of a controlled primary lesion. More favorable subgroups of oligometastases have subsequently been classified. Niibe et al. proposed the classification and naming of sync-oligometastases and oligo-recurrences [3]. Sync-oligometastasis indicates a state of oligometastases with active but controllable primary lesions. This classification appears reasonable.

Based on a review of the literature, we propose a more detailed classification of metastases and recurrence.  Table 1 shows the Niibe-Onishi-Chang classification, which includes not only oligometastases, but also polymetastases. Oligometastases and oligo-recurrences usually offer a better prognosis than polymetastases. However, oligometastases and oligo-recurrence are cancer- and organ-specific. The appearance status of oligometastases or oligo-recurrence is thus sometimes equivalent to polymetastases such as in pancreatic cancer, sarcoma, or malignant melanoma, although the last one is related to the abscopal effect, a key cure-related phenomenon for oligometastases and oligo-recurrences [5–7]. Among oligometastases and oligo-recurrences, patients with 1-2 metastases and recurrences reportedly show better prognosis than those with 3–5 metastases and recurrences [8]. In oligo-recurrence of NSCLC in only the brain or adrenal gland, patients achieve favorable survival [4, 9]. Lung or liver metastases of colon or rectal cancer are also be associated with favorable survival [10, 11]. Patients with oligo-recurrence of renal cell carcinoma also achieve long-term survival [12]. In sync-oligometastases of NSCLC affecting only the brain or adrenal gland, patients reportedly achieve relatively favorable survival [9, 13]. In sync-oligometastases of colon and rectal cancer, renal cell cancer also reportedly shows relatively favorable survival [10–12]. In oligo-recurrence of breast cancer, patients are reported to achieve relatively favorable survival [14, 15]. Niibe et al. reported that all seven of breast cancer patients with bone-only oligo-recurrence were still alive at the last followup (median followup, 40 months). In sync-oligometastases of SCLC, several cases have been reported to survive more than 5 years [16, 17]. Patients with metastatic pancreatic cancer, sarcoma, or melanoma reportedly display unfavorable outcomes [18–20]. 

This new classification, the Niibe-Onishi-Chang classification, should be revised in the future due to the rapid improvements being achieved in local and systemic therapies for cancer. This classification is tentative, but is very important given the fact that even a decade ago, many oncologists considered patients with metastasis and recurrence in only a single group.

*Yuzuru Niibe* *Yuzuru Niibe* *Joe Y. Chang* *Joe Y. Chang* *Hiroshi Onishi* *Hiroshi Onishi* *Joseph Salama* *Joseph Salama* *Takao Hiraki* *Takao Hiraki* *Hideomi Yamashita* *Hideomi Yamashita*

## Figures and Tables

**Table 1:** Niibe-Onishi-Chang classification.

Favorable	Intermediate		Unfavorable
	Relatively favorable	Relatively unfavorable
*Oligorecurrence* Site no. 1-2 NSCLC (brain and adrenal gland) Colon and rectum cancer (lung and liver) Renal cell cancer	*oligo-recurrence* site no. 1-2 breast cancer (bone, lung, and liver) SCLC (brain) site no. 3–5 NSCLC (brain and adrenal gland) colon and rectum cancer (lung and liver) renal cell cancer	*oligo-recurrence* site no. 3–5 breast cancer (bone, lung, and liver) SCLC (brain)	*Oligometastases* and *oligo-recurrence* pancreatic cancer (any site) melanoma (any site) sarcoma (any site)
	* sync-oligometastases* site no. 1-2 NSCLC (brain and adrenal gland) colon and rectum cancer (lung and liver) renal cell cancer	*sync-oligometastases* site no. 3–5 NSCLC (brain and adrenal gland) colon and rectum cancer (lung and liver) breast cancer (bone, lung, and liver)	*polymetastases *
